# Editorial: Lipid metabolism in obesity

**DOI:** 10.3389/fphys.2023.1268288

**Published:** 2023-08-31

**Authors:** Anna Maria Giudetti

**Affiliations:** Department of Biological and Environmental Sciences and Technologies, University of Salento, Lecce, Italy

**Keywords:** lipids, metabolism, obesity, diabetes, non-alcoholic fatty liver disease

The Western diet is a dietary pattern characterized by an elevated intake of high-calorie prepackaged foods rich in sugar, saturated fat, and salt. Lifestyle transition from a traditional to an industrialized/Westernized diet has dramatically increased the prevalence of overweight and obesity in recent years (Kopp, 2019; *Diabetes Metab Syndr* Obes). In obesity, white adipose tissue undergoes massive expansion due to progressive hyperplasia and hypertrophy of adipocytes, with continuous remodeling and functional alterations during disease progression. Obesity is often associated with insulin resistance, lipid metabolism alterations, and metabolic syndrome. Obesity-associated dyslipidemia presents elevated plasma levels of triacylglycerols, VLDL-cholesterol, and low HDL-cholesterol values (Vekic et al., 2023 *Current Obesity Reports*). The increased risk of cardiovascular disease in obese patients is partially explained by this dyslipidemia. Studies in both humans and animals show that metabolic alterations in adipose tissue led to a reduction in lipid storage capacity and ectopic accumulation of fat in the liver, pancreas, or muscles. Lipid accumulation in the liver not associated with alcohol abuse is referred to as non-alcoholic fatty liver disease (NAFLD) and represents the initial form of metabolic impairment which may be followed by more severe forms of hepatic impairments (Pei et al., 2020 *Biomed Res Int.*). NAFLD is a very common liver disorder and represents Western countries’ most common chronic liver disease. Fatty acids absorbed from blood, deriving from the endocytic recycling of lipoprotein remnants or *de novo* synthesis are major contributors to triacylglycerol synthesis in the liver. Alterations in any of these processes can lead to hepatic steatosis. *De novo* lipogenesis is a metabolic pathway in which fatty acids are synthesized from excess dietary carbohydrates. In this condition, most of the newly synthesized fatty acids are esterified and stored as triacylglycerols. AMP-activated protein kinase (AMPK) plays an important role in the regulation of *de novo* lipogenesis in the liver and is considered one of the key proteins capable of maintaining the cellular balance of lipid metabolism (Ferreira et al., 2023). By phosphorylating acetyl-CoA carboxylase, a key enzyme in fatty acid synthesis, AMPK can inactivate this enzyme and thus fatty acid biosynthesis. Furthermore, AMPK activation may also reduce lipogenesis by blocking the nuclear translocation of SREBP1c, a transcription factor that regulates lipogenic gene expression. Studies have shown that AMPK activation can inhibit fatty acid and cholesterol synthesis and at the same time increase the expression of genes involved in fatty acid oxidation and lipid breakdown. Thus, activation of the AMPK signaling pathway is a potential therapeutic target for liver disorders characterized by excessive lipid accumulation. Most natural AMPK agonists have been shown to ameliorate NAFLD. The key role of the AMPK pathway has been extensively reviewed by Fang et al. who performed a detailed description of each signaling axis of the AMPK pathway, its mechanism of action, and therapeutic significance. After a description of AMP-activated protein kinase structures, they described the Sirt1-liver kinase B1-AMP-activated protein kinase, AMP-activated protein kinase-ACC-carnitine acyltransferase1, AMP-activated protein kinase-SREBP-1c-lipin and AMP-activated protein kinase-mammalian target of rapamycin axes. Moreover, the authors report the status of the research on drug treatment of fatty liver targeting the AMPK pathway.

NAFLD may also be the result of the imbalance between lipid synthesis/intake and mitochondrial oxidation, especially when the latter becomes insufficient to normalize lipid levels. Mitochondria are dynamic organelles that play a key role in energy metabolism. The measurement of the oxidative capacity of the mitochondria could give an idea of the movement of lipids in the liver. However, methods to estimate human liver mitochondrial activity are lacking. The study from Mucinski et al. collaborators describes a non-invasive breath test to quantify complete mitochondrial fat oxidation and how the test results changed when the liver disease state is altered over time. The work, unlike comparable studies that used labeled octanoate, also evaluated contemporaneously liver glucose metabolism. In the work, enrolled patients with suspected NAFLD underwent a diagnostic liver biopsy and were assigned histological scores using the NAFLD activity score. Thus, labeled octanoate, a medium-chain fatty acid was furnished orally, and the breath samples were collected over time by measuring ^13^CO^2^ by mass spectrometry. Despite the need to enlarge the cohort of enrolled patients, the study demonstrated that in subjects with NAFLD, the oxidation of orally-administered octanoate can be associated with hepatic glucose tolerance and changes in liver fat and glucose production. Thus, this test seems to be promising as an indicator of progression or changes in liver fat content in patients with NAFLD.

Patients with NAFLD are generally asymptomatic and liver biopsy represents the gold standard for NAFLD diagnosis and staging. However, this is invasive, costly, and not without risk. Biomarkers that could diagnose and stage disease would reduce the need for biopsy and allow stratification of patients at risk of progression to non-alcoholic steatohepatitis (NASH). A pilot study by Watt et al. and collaborators reports the analysis, conducted by high-sensitivity cytokine array I, immunoassays, and ELISAs, of serum cytokines from controls, benign simple steatosis, NAFLD/NASH, and alcoholic liver disease patients. The authors investigated the pattern of 20 individual biomarkers and find that thirteen out of 20 were significantly different among groups including IFNγ, EGF, IL-1β, IL-6, IL-8, IL-10, TNFα, FABP-1, PIIINP, and ST2/IL-33R, albumin, AST, and ALT. Moreover, five of these significantly changed biomarkers were identified for further investigation. The study allows concluding that TNFα may be a useful biomarker for staging patients with early liver disease (simple steatosis), and ST2/IL-33R levels may correlate with the extent of liver fibrosis deposition and disease progression. Thus, the use of these biomarkers, in association with known clinical risks for NAFLD could potentially allow patient stratification into “low” and “high” risk of disease progression. Then, biomarker combinations, more than single biomarker consultation, may help stratify risk and stage disease where patients are averse to biopsy.

Muscle plays an important role in obesity-associated dyslipidemia and type 2 diabetes. After a meal, the insulin secreted can induce the entry of more than 70% of the circulating glucose into the muscle, where glucose is stored in the form of glycogen. Accumulation of muscle-skeletal lipids can be often observed in obesity. This condition alters the physiology of the muscle inducing the inability to properly respond to insulin signals, a condition known as insulin resistance, with increased glycemia and diabetes occurrence. Exercise is an effective strategy to prevent and treat obesity and its related comorbidities. Indeed, exercise induces significant body fat mass loss, optimizes energy expenditure, and improves hypothalamic circuits controlling appetite-satiety. Moreover, exercise ameliorates insulin resistance and induces the secretion of soluble factors, including myokines that have been shown to play an essential role in regulating muscle metabolism. Among myokines, interleukin 6 (IL-6) is closely associated with skeletal muscle contraction. IL-6 participates in various inflammatory responses, including non-classical inflammatory responses such as adipogenesis. It has been demonstrated that concentrations of IL-6 increased in response to exercise and play a key role in mobilizing fat from adipose tissue thus furnishing substrates for contraction at times of low glucose availability. Thus IL-6 has a catabolic function in fat metabolism and blocking endogenous IL-6 signaling promotes storage of fat.

By secreting IL-6, skeletal muscle can induce lipolysis of adipose tissue, thus obtaining energy for contraction. The role of IL-6 in muscle contraction and lipid metabolism was by Lin et al. who discussed the role of IL-6 in the process of muscle contraction through three mechanisms of IL-6 signaling, namely, classic, trans, and cluster signaling. Moreover, the work reviewed the paracrine role of skeletal muscle-derived IL-6 in lipid metabolism, insulin resistance, and glucose tolerance providing valuable insights into the regulatory function of skeletal muscle-derived myokines in lipid metabolism. The work addresses an important Research Topic regarding the interaction between skeletal muscle and adipose tissue through muscle paracrine signaling providing insights into the treatment of diseases associated with ectopic fat deposition.

Collectively, articles comprising this Research Topic provide new opportunities for the diagnosis and progression assessment of obesity-related diseases such as NAFLD, skeletal muscle insulin resistance, and type 2 diabetes. Furthermore, they collect information currently available on the key regulators of lipid metabolism in muscle and liver thus providing the starting point for the development of new therapeutic options for the prevention and treatment of metabolic diseases associated with obesity.
